# Breathing Life Into Meta-Analytic Methods

**DOI:** 10.5334/joc.389

**Published:** 2024-07-22

**Authors:** David Allbritton, Pablo Gómez, Bernhard Angele, Martin Vasilev, Manuel Perea

**Affiliations:** 1 DePaul University, Department of Psychology, Chicago, IL, USA; 2 Skidmore College, Saratoga Springs, NY, USA; 3 CSUSB –Palm Desert Campus, Palm Desert, CA, USA; 4 Universidad Nebrija, Madrid, Spain; 5 Bournemouth University, Poole, UK; 6 University College London, London, UK; 7 Universitat de València, Valencia, Spain

**Keywords:** meta-analysis, app, Bayesian statistics, meta-analysis updating

## Abstract

Meta-analyses have become indispensable in the behavioral sciences, combining and summarizing data from multiple studies. While they offer many advantages (e.g., increased power, higher generality, and resolving conflicting findings), they currently only provide a snapshot at a given point. In active research areas, frequent meta-analytic updates are necessary to incorporate new evidence. We propose guidelines for live, dynamic meta-analyses and introduce an accessible tool using the R environment. Our app, powered by the Shiny package, enables the meta-analyst to integrate evidence interactively as an update of an existing meta-analysis or from scratch (i.e., a new meta-analysis). By embracing dynamic meta-analyses and leveraging modern tools, researchers can ensure up-to-date meta-analyses in their respective fields.

Meta-analysis, a term first coined by Glass (1976), is a powerful statistical technique that integrates findings from individual studies, providing more precise estimates of effects and deepening our understanding of research questions by synthesizing evidence (see Egger et al., 2022 for a historical overview). In 1976, Glass observed a notable surge in the number of studies being conducted, and since then, this growth has further gained momentum. Recent estimates suggest that the volume of research papers doubles every 12 to 17 years (Bornmann et al., 2021; Fire & Guestrin, 2019). This dramatic increase is evident when comparing the hundreds of meta-analysis papers in the 1980s (Borenstein et al., 2009) to a staggering 13,000+ papers in 2020 alone, as revealed by a PsycINFO search targeting titles, abstracts, and keywords.

A meta-analytic paper can be considered a snapshot of a family of studies taken by the authors. Like any family picture, this snapshot is frozen in time, reflecting the inclusion vs. exclusion choices made by the photographer. There is no doubt about the key role of meta-analytic papers in science; indeed, they tend to have high visibility; however, a reader of such papers must contend with at least two key issues. The first is that new studies are likely to be published after the publication of the meta-analytic paper. For instance, Shojania et al. (2007) estimated that, for active areas of medical research, 7% of meta-analyses missed important findings on the day of publication, while 23% of meta-analyses required updating only two years after their publication. Furthermore, as a field evolves over time, the effect size estimates can change in specific directions. This can be due to a more sophisticated methodology but also a change in the direction of publication biases (e.g., see Sanchez-Azanza et al.’s 2017, bibliometric analysis of the bilingual advantage literature). The second issue is that there could be reasonable disagreement on what studies should be used or even what criteria should be used to select studies. Indeed, it is not uncommon to have meta-analyses on the same topic with differing conclusions (see Paap et al., 2020 for discussion).

To address the two issues outlined above, we present here a tool that turns snapshots into a meta-analytical living method. The idea of a “living systematic review” that could be updated electronically was first proposed by Chalmers (1986) in the context of establishing the Oxford Database of Perinatal Trials. In clinical research, there have been many attempts to use databases to provide a more up-to-date view of a particular area (e.g., see Elliott et al., 2014). Most recently, during the COVID-19 pandemic, several living systematic reviews were created to compile the evidence available on the efficacy of different treatments (Elliott & Turner, 2022). Despite these advances, living systematic review and meta-analysis techniques are still rare in less well-resourced research areas (apart from the research mentioned above), particularly those not benefiting from large trial database projects. This is due to the technical challenges of implementing them up to now.

Given this, breathing life into meta-analysis is in order, following the example set by the large clinical databases. We want to provide researchers with a tool that enables a dynamic exploration of the phenomena under scrutiny. The present article presents a novel, effective, and accessible approach to deploying dynamic meta-analyses. The basic idea is that, unlike at the time of Glass’ (1976) seminal work, we have at our disposal novel tools that allow information to be updated quickly (see the examples of prior work below), and, unlike at the time of Chalmer’s (1986) first suggestion of a frequently updated electronic database, these tools are now available to researchers outside of large clinical database projects. Being able to update the analysis continually is a crucial improvement to the way we present meta-analyses. We begin with a set of goals that transform meta-analysis into a living tool that can be deployed by any community of scientists around the world, even with limited resources; then, we present an example of such a tool in action, and we end with suggestions and possibilities for additional analyses and guidelines on how dynamic meta-analyses should be implemented.

## What should a meta-analysis tool that is alive look like?

In various scientific fields, concerted efforts have established guidelines for reporting and implementing meta-analyses. One prominent example is the PRISMA statement (Moher et al., 2009), which provides minimum reporting guidelines initially intended for clinical research. Additionally, numerous tutorials cater to specific audiences, such as those focusing on statistical packages (e.g., Berkhout et al., 2023; Viechtbauer, 2010) or variations of the method (e.g., Rouder et al., 2019). Interested readers can readily access these guidelines, and we recommend that readers consult relevant guidelines when constructing a new meta-analysis and systematic review (e.g., Moher et al., 2009; Page et al., 2020). This paper introduces a user-friendly tool designed to transform meta-analysis into a dynamic, evolving process. To achieve this, we focused on creating a tool endowed with multiple desirable features:

### Availability

To ensure widespread accessibility, the tool must be freely available and open source. We have developed the tool within the Shiny app environment to achieve this objective. Shiny apps (Chang et al., 2023) are web-based interactive applications created using the R programming language and the Shiny framework. This platform enables R users to design and distribute interactive data visualizations and analyses on the web, fostering collaboration and knowledge sharing among researchers. Importantly, although we use Shiny to achieve the goals of availability and open source, these goals can also be achieved on other platforms.

Another dimension of availability is having a low technical barrier to entry. The tool presented here allows researchers to carry out living Bayesian meta-analyses without any need to program, modify, or even download or host the Shiny app, which was our goal. Importantly, should researchers want to change and augment the app’s capabilities, they can do so as the code is freely available within a widely used Shiny platform.

### Study Selection

One of the most contentious issues in meta-analytic work is the selection of studies to be considered in the meta-analysis. Researchers must contend with publication biases (e.g., Vevea & Hedges, 1995) and with the intrinsic difficulty of any meta-analysis method: defining a set of studies requires a theoretical position (e.g., Field & Gillett, 2010). This definition can become a contentious issue because it reflects a tension: the only way to select the optimal criteria for inclusion/exclusion is to fully understand the phenomena at hand; conversely, the only way to understand the phenomena at hand is to include all the relevant studies. This circularity forces researchers to make somewhat subjective choices; this problem was already identified by Fisher (1955; see also the response by Pearson, 1955) in the context of sampling and is connected to Venn’s (1876) reference class problem.^1^ Hence, researchers might want to examine the robustness of findings by recalculating the analysis incorporating a different set of studies and assumptions. This practice is gaining popularity, termed *sensitivity analysis* (Patsopoulos et al., 2008). Continuing with the family photo analogy, the photographer needs to have a model of the family dynamics to decide if a second cousin should be included in a wedding picture. The decision-maker needs to understand the family dynamics to make an informed choice.

### Adding studies

As stated above, the current structure of meta-analysis papers is inherently static. To advance the methodology, future meta-analyses should be designed to enable researchers to add studies over time, fostering community-based collaborations. Furthermore, publication bias and, specifically, the file drawer problem, which can lead to null results being excluded from published meta-analyses (Rosenthal et al., 1979), could be addressed by incorporating unpublished results that may become available for inclusion in dynamic meta-analyses. As outlined above, the decision to include or exclude these results should be left up to the researcher. The potential for researchers to collect and utilize additional data in meta-analyses beyond the published literature is an important feature of a living meta-analysis.

### Outputs

While Forest plots are canonical to visualize meta-analyses (Higgins et al., 2009), other outputs, such as funnel plots and posterior distributions, should also be available.

#### Inference philosophy

One can perform and interpret meta-analyses with frequentist and Bayesian methodologies. There are compelling reasons to prefer the Bayesian approach (see also Berkhout et al., 2023) because it is particularly well-suited for a living meta-analysis as it makes prior beliefs and the impact of new evidence explicit. Our tool allows Bayesian meta-analysis, but because we privilege speed in the current version, we provide the frequentist method as a default. Note that there is a non-trivial processing cost of performing Bayesian meta-analysis. Hence, we prefer the faster frequentist methods as researchers explore the tool, so we have implemented that as a default.

##### Prior related work

During the conceptualization phase of this work, a group of researchers in physiology (Wolf et al., 2021) published a tool that fulfills many of the requirements outlined above. Their work has proven to be an excellent starting point for our efforts. In their work, they explored the relationship between transcutaneous auricular vagus nerve stimulation (taVNS) and vagally mediated heart rate variability (vmHRV), and they concluded that there is no support for the hypothesis that HRV is a robust biomarker for acute taVNS.

Wolf et al.’s paper has made a significant impact in physiology, garnering 49 citations as of winter 2024. However, we contend that their most valuable contribution lies in the realm of meta-analysis. They introduce a novel approach called a “living meta-analysis” through a Shiny R web application (https://vinzentwolf.shinyapps.io/taVNSHRVmeta/). This application utilizes the bayesmeta package (Röver, 2020) to generate outputs even with a minimal dataset of four studies. Furthermore, it incorporates Bayes Factors (BF), a popular form of statistical inference in the cognitive sciences (i.e., BFs offer a measure of how likely the data is given Model 1 [e.g., alternative hypothesis] relative to how likely the data is given Model 0 [e.g., null hypothesis]). In addition to BFs, their application provides a comprehensive set of outputs, including a summary of the posterior distribution, a robustness check for BFs, and various informative graphs such as outlier checks, forest plots, funnel plots, and plots of the prior and posterior distributions.

The app’s key feature is its interactive functionality, empowering users to modify the model priors and data selection parameters and observe the consequent impact on meta-analytic results. This capability enables users to make informed decisions based on numerous factors. For instance, they can select the study design (between vs. within) and specify the type of blinding employed. Furthermore, users can choose the type of control and adjust specific sample characteristics. By offering these options, the app enhances the user’s ability to customize and tailor the meta-analysis according to their specific needs.

The approach employed by Wolf et al. not only ensures the accessibility and transparency of data and analysis, aligning with the principles of open science, but it also facilitates sensitivity analyses by enabling users to make real-time modifications and observe the corresponding outcomes. This immediate feedback loop enhances the flexibility and exploratory nature of the analysis.

However, it is important to note a key limitation of their approach: any re-analysis conducted by users is confined to the existing dataset provided by the original authors. Although users are encouraged to contact the authors and suggest new studies for potential inclusion, their current app only allows them to add new studies to update the results. Thus, while the app allows for dynamic exploration, its scope for incorporating new evidence is restricted. Indeed, as of late 2023, there have not been any updates on the original dataset.

One of our primary objectives in expanding upon Wolf et al.’s tool was to effectively provide users with the capability to interactively and rapidly incorporate new studies into an existing meta-analysis and observe the resulting outcomes. We aimed to enhance the tool’s functionality to seamlessly integrate additional evidence. Furthermore, we set out to achieve a more ambitious goal. We have developed a tool that enables users to create a Bayesian meta-analysis using a dataset that can be either individually created or crowdsourced. By empowering users to conduct extensions and independent analyses, we aimed to enhance the versatility and applicability of the tool in a wide range of research scenarios.

Researchers in cognitive psychology have made significant strides toward crowdsourcing data using Shiny platforms. For instance, Buchanan et al. (2022) have created a Shiny app to track participants and make data available in multi-lab experiments (e.g., https://psa007shiny.psysciacc.org/tracker/). While the Buchanan et al. app does not aim to analyze data, it reflects the *Zeitgeist* of collaborative multi-site data sharing that influences our proposal.

## Advancements and Innovations in Living Meta-analysis 2.0

In our work, we built upon the foundation provided by Wolf et al. (2021), utilizing their *bayesmeta* analysis modules and adopting their input panel for selecting model priors. We also retained most of their output panels, including the outlier test, forest plot, funnel plot, and tables of model statistics, as they displayed the results effectively. More importantly, we introduced new features to enhance the functionality and reproducibility of the app.

The first significant innovation in our app version can be found in the “Study Criteria” tab of the user interface. Instead of being entirely static with predefined selection options, this tab dynamically generates components based on the contents of the uploaded data file. Whenever a new data file is uploaded, a matching “Study Criteria” tab is automatically generated, adapting the app to the updated dataset.

The second major innovation was the ability for users to upload new data. This can be done by replacing the currently loaded data file with an Excel spreadsheet or adding individual data points through a user-friendly interface generated in real-time. These advancements empower users to conduct meta-analyses on a wide range of phenomena across many fields, taking advantage of the flexibility of our tool.

Furthermore, we incorporated additional panels in the app’s user interface, such as panels for frequentist analyses, a display of the currently active data file, and two panels for downloading essential information required for reproducibility. One download panel allows downloading the data itself, input selector settings, R function calls, and model parameters that generate the currently displayed results, and the second panel allows downloading R Code or R Markdown that can reproduce the displayed analyses. By offering these features, we aim to enhance transparency and reproducibility in the meta-analysis process.

When combined, all these innovations allow our tool to be used for a meta-analysis on any field of science with quantitative data.

## Description of our App

The description of the app has two sections. In the first section, we describe the app’s capabilities using a sample meta-analysis uploaded as a default. To that end, we employ a recent meta-analysis published by Vasilev et al. (2018). In the second section, we provide instructions on how to upload new data for different phenomena. This second part is, of course, the most significant innovation.

### Default analyses

**Figure 1:**
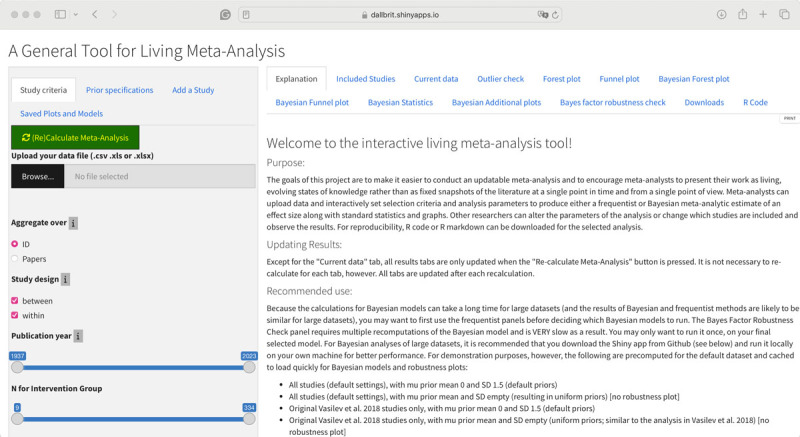
The Input sidebar is on the left side, and the output is on the right as tabs.

Our tool running on shinyapps can be found at https://dallbrit.shinyapps.io/Breathing_Life_into_MetaAnalysis/ (see Figure 1 for the opening screen). As a default and to highlight some of the capabilities, we have populated the data with an updated version of the Vasilev et al. (2018) meta-analysis on the effect of noise on reading. In the published meta-analysis, Vasilev et al. included 54 studies with reading comprehension accuracy as a dependent variable. For the updated version, we added some newly published studies and new unpublished data, resulting in 79 studies.

The app has two sections: a sidebar panel for inputs (left side of Figure 1) and a Main Panel for displaying outputs (right side of Figure 1). In the sidebar input panel, there are four tabs:

#### Study Criteria

**Figure 2:**
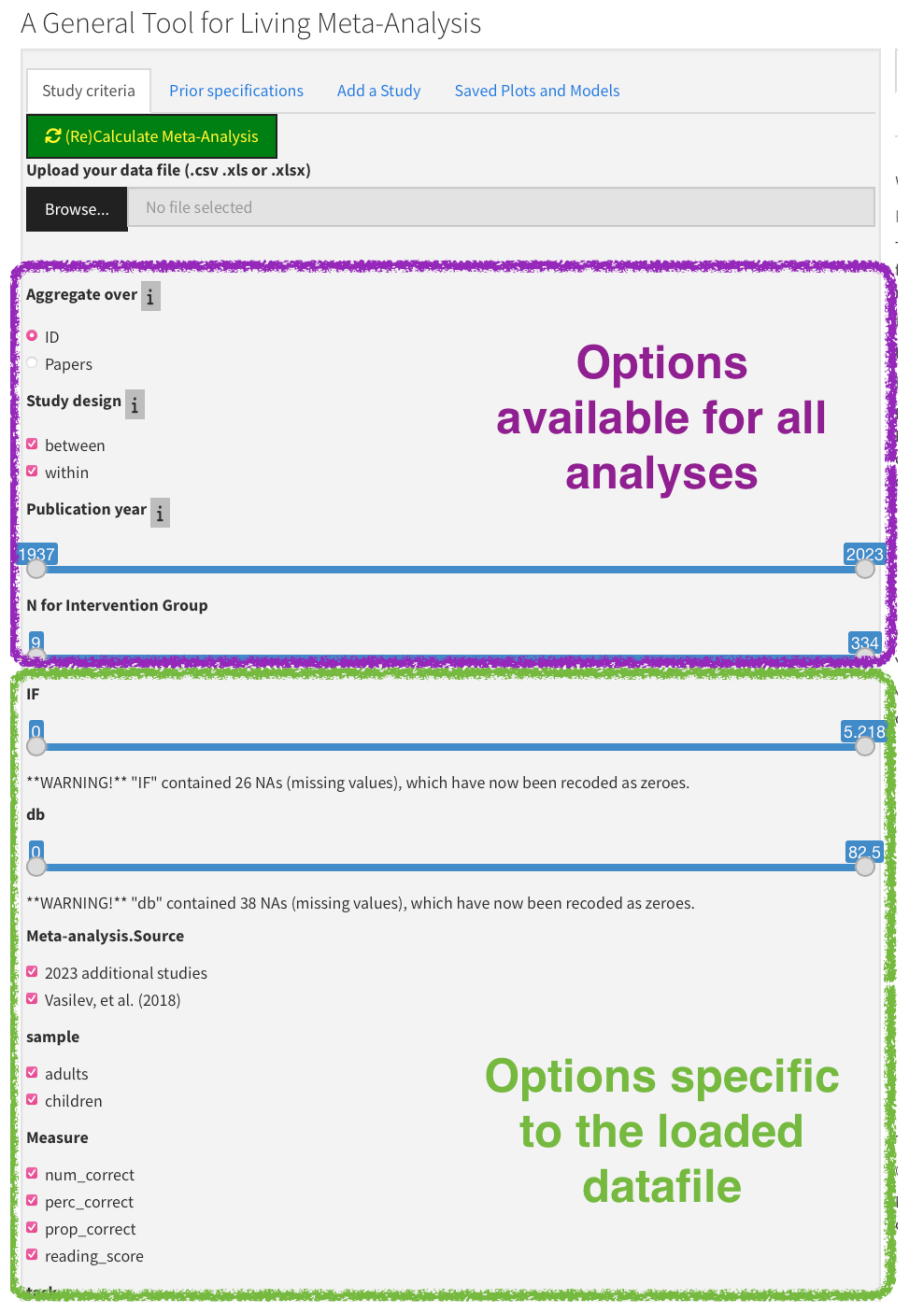
Options in the study criteria tab. The options within the top square are general for all analyses, and the ones in the bottom square are specific to the data file being used; in this case, it is for the updated Vasilev et al. meta-analysis.

Users can choose the aggregation level either at a paper level or an ID level. Note that the ID is simply a column in the spreadsheet that uniquely identifies a row, which could be a subset of data or an Experiment or sub-Experiment in a paper. Users can select the design of the studies to be included in the analysis (between, within, or both), the range of publication years, and the range for N in the intervention group. These options are available for all meta-analyses (see Figure 2). The remaining options are dependent on the data file used (see the **Upload your data file** section below) and have three sections: the categorical factors analyzed or manipulated in the original studies (shown as checkboxes), the numeric values of factors analyzed or manipulated in the original studies (shown as numeric slider inputs), and the studies included (shown as checkboxes). The user can use these three sections to expand and constrain the set of studies to be meta-analyzed. Note that for the default dataset the filters refer to the variables included in the sample data file (e.g., dB as in the loudness of the sounds in the sample dataset). Also, if studies are included but do not include data for a selection variable, there is a warning message stating that the NAs are turned to zeros. This is the default action in the metanalysis function.

#### Prior specification

**Figure 3:**
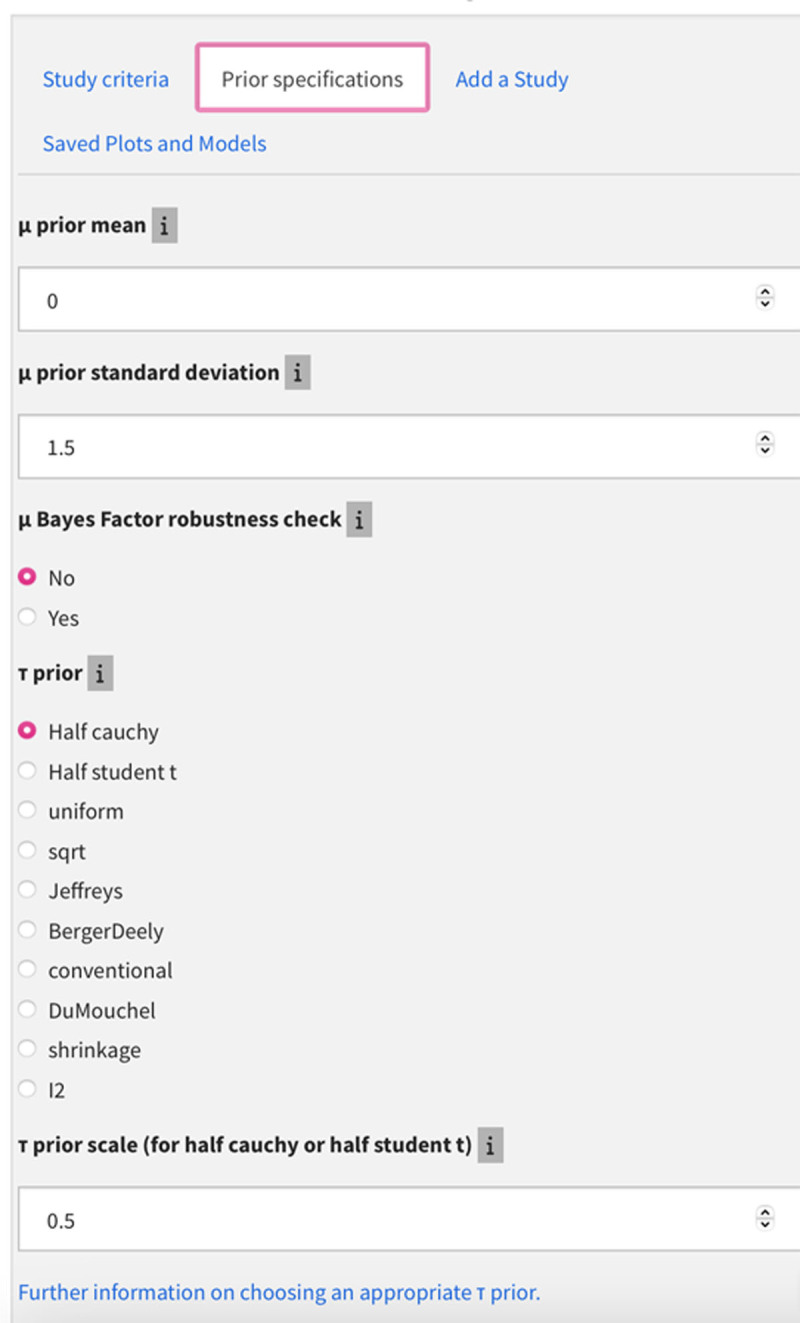
Prior Specification tab.

In this tab (see Figure 3), users can change the features of the prior distribution by specifying the mean and standard deviation of the *mu* parameter and the functional form of the *tau* parameter. As is the case in most Bayesian analyses, the effect of the choice of priors on the posterior distribution depends on the amount of data available. The default priors in the app for the R bayesmeta function are those used by Wolf et al. (2021), and they can be changed in the “Prior specifications” tab (identical to the tab in the Wolf et al. app). The µ prior has a default mean of zero and a standard deviation of 1.5. To instead employ a uniform prior for µ (as did Vasilev et al., 2018), the user can empty the input boxes for the µ prior mean and SD before (re)calculating (since a uniform prior is the default for the bayesmeta function). The default prior for τ is a half Cauchy with a scale parameter of 0.5. Several alternative priors for τ can be specified in the app, including a uniform prior for τ similar to that used by Vasilev et al. (2018).

#### Add a Study

The add a study section allows users to quickly add one study at a time. This is a useful way to update the meta-analysis with new data and add a study to the data set. Note that to save all the studies considered in a meta-analysis, including added studies, the data file must be downloaded from the “Downloads” tab of the Main panel.

#### Saved Plots and Models

Whenever a Bayesian model is computed or a Bayes factor robustness plot is produced, it is cached for the duration of the session to avoid potentially lengthy delays from unnecessary re-computation. A few pre-computed models and plots are pre-loaded with the app for demonstration purposes. The user can also save models and plots as an RDS file that can be uploaded for future sessions.

In the Main panel, there are ten tabs that we describe below:

#### Explanation

There is a minimal explanation of the tool in the first tab that describes the key features of the app.

#### Included Studies

The Included studies are the subset of studies that fit the user’s selected study criteria. Only these studies contribute to the analyses when the user clicks the “recalculate meta-analysis” button.

#### Current Data

This section displays the currently updated data file (all studies, not just the “included studies”), plus any manually input studies.

#### Outlier Check

**Figure 4:**
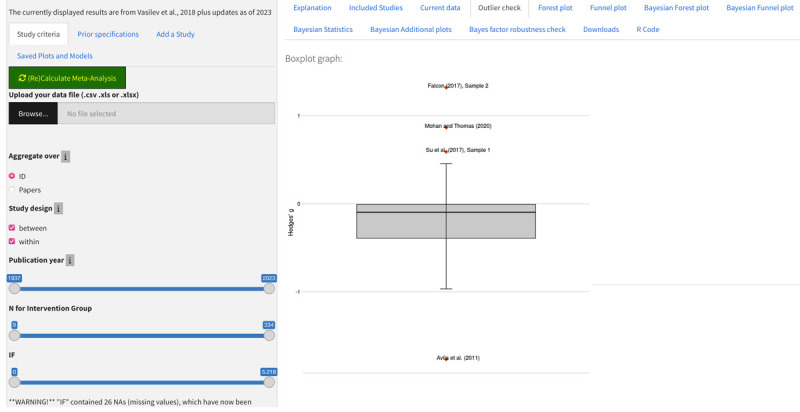
Outlier Check using the default data.

To assess the impact of outliers, a tab shows a boxplot of Hedges’ *g*. As can be observed in Figure 4, in the default data set, there are a handful of clear outliers.

#### Forest plot

A forest plot based on the frequentist random effects model of the data is displayed in this tab. Weights and 95% confidence intervals are displayed along with the model’s prediction interval, Cochrain’s Q, and I-squared.

#### Funnel plot

For further assessment of the data, a funnel plot is shown in this tab (Light & Pillemer, 1984). The funnel plot aids in detecting publication bias, and users should be aware of the appropriate use of such plots (e.g., Sterne et al., 2011).

#### Bayesian Forest plot

**Figure 5:**
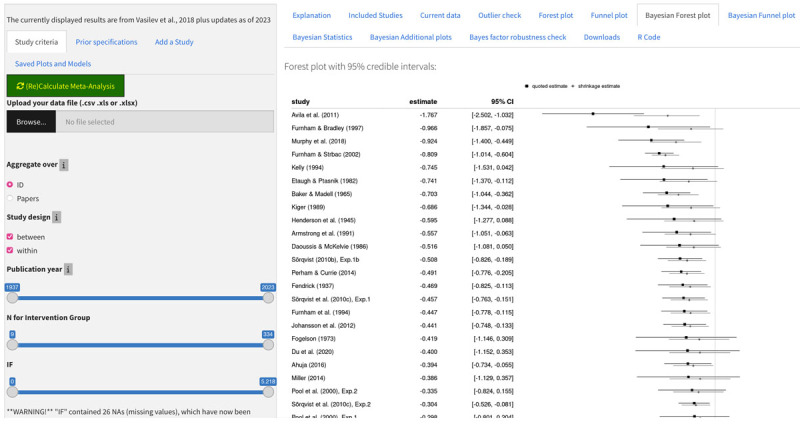
Bayesian Forest Plot using the default dataset (check the Frequentist Forest Plot first when analyzing new data).

The Bayesian forest-plot is shown under the tab of the same name. Importantly, the plot shows the 95% Credible intervals and the Bayesian shrinkage (see Figure 5). All tabs that require the computation of a Bayesian model include “Bayesian” in their title, and an alert asks the user whether to proceed whenever an output would require that a Bayesian model be computed for the first time.

#### Bayesian Funnel plot

This tab provides a funnel plot based on the Bayesian model of the data.

#### Bayesian Statistics

The statistics tab includes the Bayes factors obtained from the bayesmeta package as well as summary statistics on μ (mu, the posterior distribution of the effect), and τ (au, the posterior distribution of between-study heterogeneity). Also, the joint maximum-a-posteriori for the two parameters is presented. These statistics are the same ones that Wolfe et al. (2021) present in their work.

#### Bayesian Additional plots

**Figure 6:**
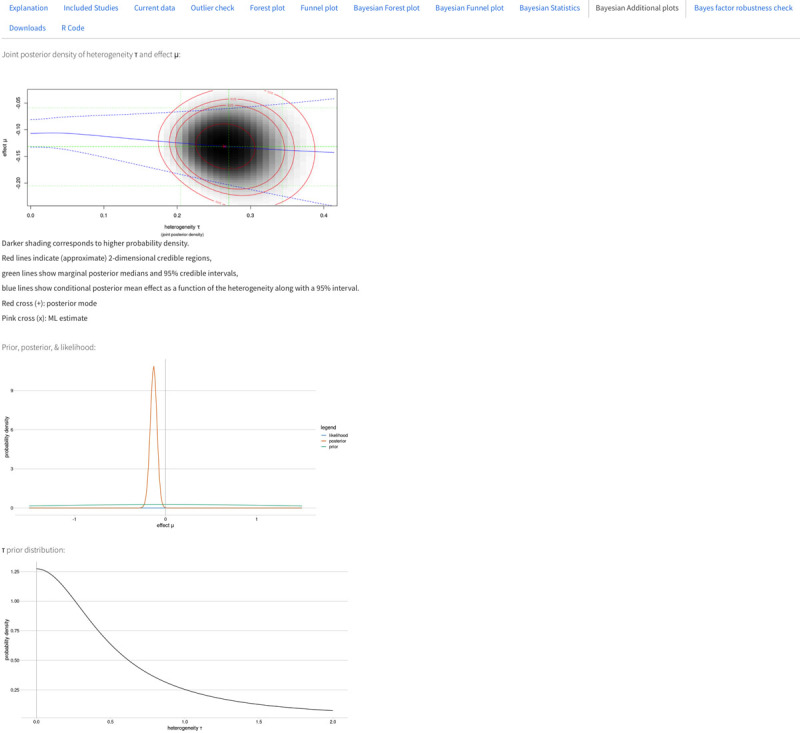
Bayesian Additional Plots tab.

Under this tab, the graphical displays of the statistics section described above are presented (see Figure 6), namely, plots of the posterior distribution and the joint posterior density of μ and τ.

#### Bayes factor robustness check

**Figure 7:**
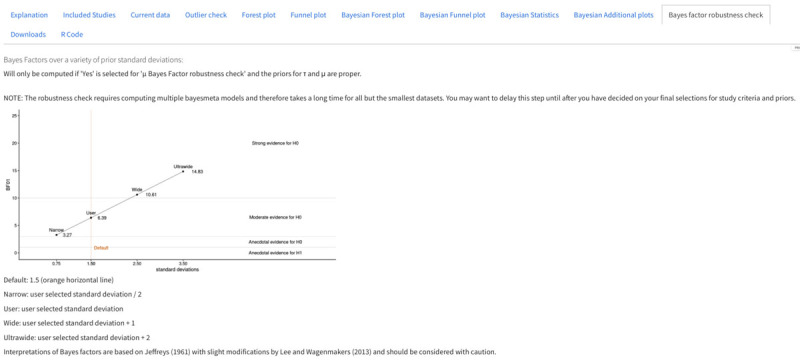
Output of the prior check. The data used in the example is a subset of the Vasilev et al. meta-analysis examining only children. Because there are only a few studies, the choice of priors has large consequences.

If the “µ Bayes Factor robustness check” option from the *Prior specification* section is chosen, a graph displaying the Bayes factors over various priors is presented. As this option is computationally intensive, it is not selected by default. On the y-axis, the BF_01_ (null / alternative) or BF_10_ (alternative / null) is displayed, depending on which hypothesis is supported. In Figure 7, we present a case in which the choice of priors plays a significant role, given the small number of papers considered in the analysis (only children).

#### Downloads

This tab allows the download of several files that may be useful for further analysis or examination of the model: (1) the data as uploaded initially, (2) the data in use in the latest analysis run, (3) a list of criteria and prior specifications, and (4) the function call. All these files are in .xlsx format, splitting the different components within the file into different sheets (tabs).

#### R Code

To further support reproducibility, this tab allows users to download R code as a regular R file or an R markdown file that can reproduce the currently displayed analyses and plots. The downloaded code includes all the user’s selections in the Study Criteria and Prior Specification tabs.

#### Updating Results

Except for the “Current data” tab, all results tabs are only updated when the “Re-calculate Meta-Analysis” button is pressed. However, it is not necessary to recalculate each tab. Tabs are updated after each recalculation, although, in some instances, Shiny generates rendered outputs to the screen only when the user accesses a panel.

**Figure 8:**
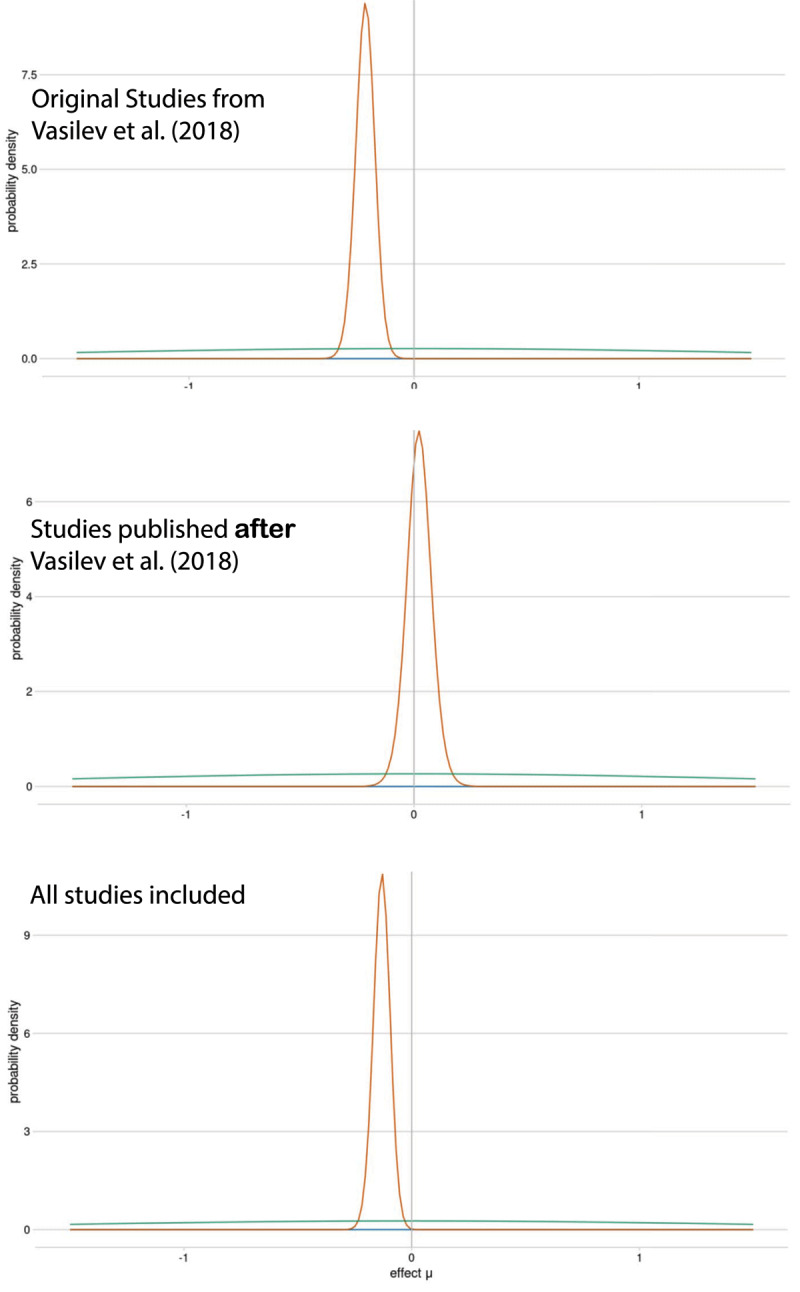
Examples of posteriors with different selection criteria; this shows type of figure shows how an effect changes over publication period.

The (Re)Calculate Meta-Analysis function can be easily demonstrated using the default dataset. If we do the meta-analysis only with the original Vasilev data set (see Figure 8), we obtain a larger µ for the posterior of the effect (mean = –0.217; shown in top Panel) than if we include the all studies (mean = –0.137; shown in bottom Panel); this was the case because there is essentially no effect in the newest results (mean effect = 0.02; shown in middle Panel).

### Uploading a new data file

The most important contribution of this tool is that it allows communities of scientists to sustain a living meta-analysis that can be updated any time a study is published or even as the meta-analytic data is collected. Meta-analysts can upload data in a csv or Excel file (of course, this includes downloading the default data as an Excel file and then modifying that file) and interactively set selection criteria and analysis parameters to produce a Bayesian or frequentist meta-analytic estimate of an effect size along with standard statistics and graphs like those described above for the default data. Other researchers can alter the parameters of the analysis or change which studies are included and observe the results, which has a potentially transformational impact on communities of researchers, as it allows anyone to re-analyze while changing assumptions and adding data from published and unpublished experiments.

**Figure 9:**
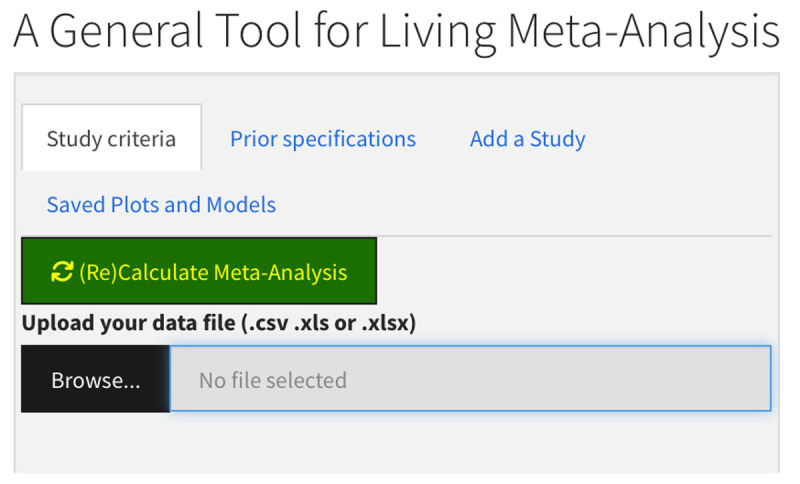
Adding a new data file.

When users upload an entirely new data file in the “Study criteria” tab (see Figure 9), it will replace the current data, including anything previously input through the “Add a Study” tab. The file to upload should be in.csv, .xls, or .xlsx formats, and the structure of the data file determines the contents of the app’s “Study Criteria” panel. It must begin with two columns labeled “yi” and “vi” containing the standardized effect size (Hedges’ *g*) and its variance. The next 11 required columns contain study design and identifying information, such as the source (“Paper”), publication year, design, and number of subjects. Following the required columns, two optional sets of columns with topic-specific information can be included. The “Selection Factors” (all columns between two empty columns labeled “Begin.Selection.Factors” and “End.Selection.Factors”) are categorical variables that are used to generate checkbox input selectors. The “Selection Numerics” (between columns labeled “Begin.Selection.Numerics” and “End.Selection.Numerics”) are used to generate slider input selectors. When a new data file is uploaded, a new “Study Criteria” panel is generated. By default, all checkboxes and sliders are initially fully selected, indicating that all studies in the data file will be included in the meta-analysis. There is one optional column for “r,” which is the within-study correlation of outcome measures for within-subject designs. This *r* estimate is only needed when manually adding a within-subjects study by entering means and standard deviations instead of entering pre-computed effect sizes. In such cases, a new variable labeled “r” will be created if no such column already exists in the data file. Additional columns can exist but will be ignored by the program.

The “Add a study” tab allows the user to manually input one or more new effect sizes, either to include new studies that were discovered after the data file was created or to run “what if” analyses to see how the meta-analysis results might change. The user can enter an effect size (Hedges’ *g* and variance of *g*) directly, the same as required when uploading an Excel file. In doing so, the user will have already made all of the necessary decisions about whether to include both within-subjects and between-subjects effect sizes in the same meta-analysis and about how to best calculate within-subjects effect sizes so that they will be comparable to between-subjects effect sizes (see, for example, Harrer et al., 2021, Section 3.3.1.3 for a discussion of the issues involved.). A second option for manual input is for the user to provide *d*, the variance of *d*, and *N* instead of providing *g* and the variance of *g*. In that case, the app will calculate *g* and the variance of g. It will assume that any adjustments for comparability of between-subjects and within-subjects effects are already reflected in the user-provided values of *d* and the variance of *d*. A third option for inputting new data points is for the user to provide means, standard deviations, and *N*, letting the app calculate the new effect size (using *escalc* function from the *metafor* package in R, Viechtbauer, 2010). This is probably the easiest option since that information is likely to be readily available for a new study, but the user should note carefully the “Calculation methods” section of the app’s “Explanation” tab, which specifies how an effect size will be calculated depending on the type of design the user specifies (between vs. within) and the specific means and SDs the user provides. Users are advised to consult the documentation of the *metafor::escalc* function to ensure that the app’s calculated effect sizes are consistent with the methods used to calculate effect sizes for the studies in the original uploaded data file.

Importantly, when using other files, one must acknowledge a wide range of reporting practices in meta-analyses. We found that older papers, from before 2015, often do not include a link to a data set, and even more recent papers might not present all the relevant data. For example, Gunnerud et al. (2020) did not provide the number of subjects per group. Instead, they provided a binary variable with one value for N < 50. The other value for studies with N > 50. In other data sets, like Aksayli et al.’s (2019), they only report the total number of subjects and not the number in each group (in many cases, the assumption of equal Ns could produce usable analyses). While this landscape is not ideal, the emergence of tools like this one might encourage authors, reviewers, and editors to avoid minimalist datasets.

To exemplify the uploading of new data, we use Maldonado et al.’s (2020) dataset. In this database, the authors perform a meta-analysis of aging effects in various tasks. We renamed and reorganized the columns. For demonstration’s sake, we also made some assumptions to make the data fit the requirements of the app; namely, in some studies, the mean age of the participants was not provided, and instead, a range of ages was reported; in these cases, we simply use the midpoint of the range.

Below are some examples of the analyses done with this new dataset. We carried out three meta-analyses to highlight the tool’s capabilities: We analyzed the studies before and after 2005 and selected only the lexical and reading studies in all cases. Note that these analyses are strictly for demonstration, and any theoretical claim is beyond the scope of the present manuscript.

#### Studies before 2005

**Figure 10:**
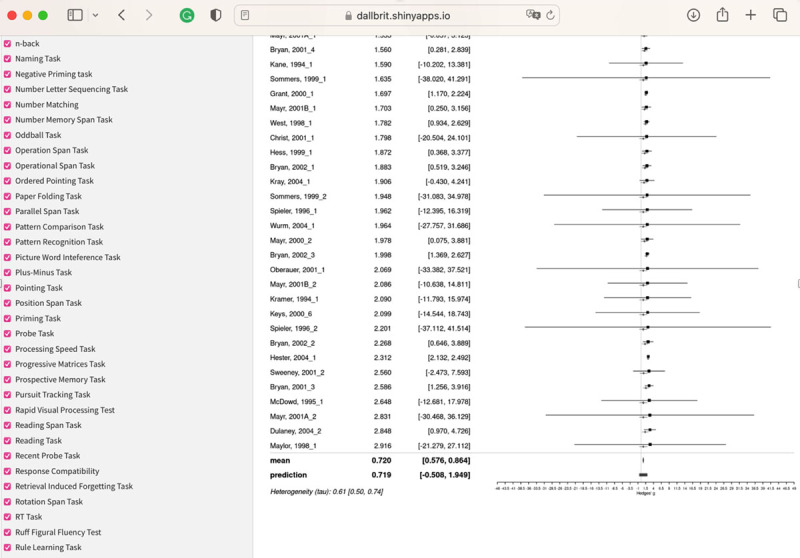
Forest plot of the papers from before 2005 in the Maldonado dataset.

There are 154 studies between 1993 and 2005, and the mean for the posterior distribution is 0.72. Figure 10 shows the forest plot for this analysis.

#### Studies after 2005

**Figure 11:**
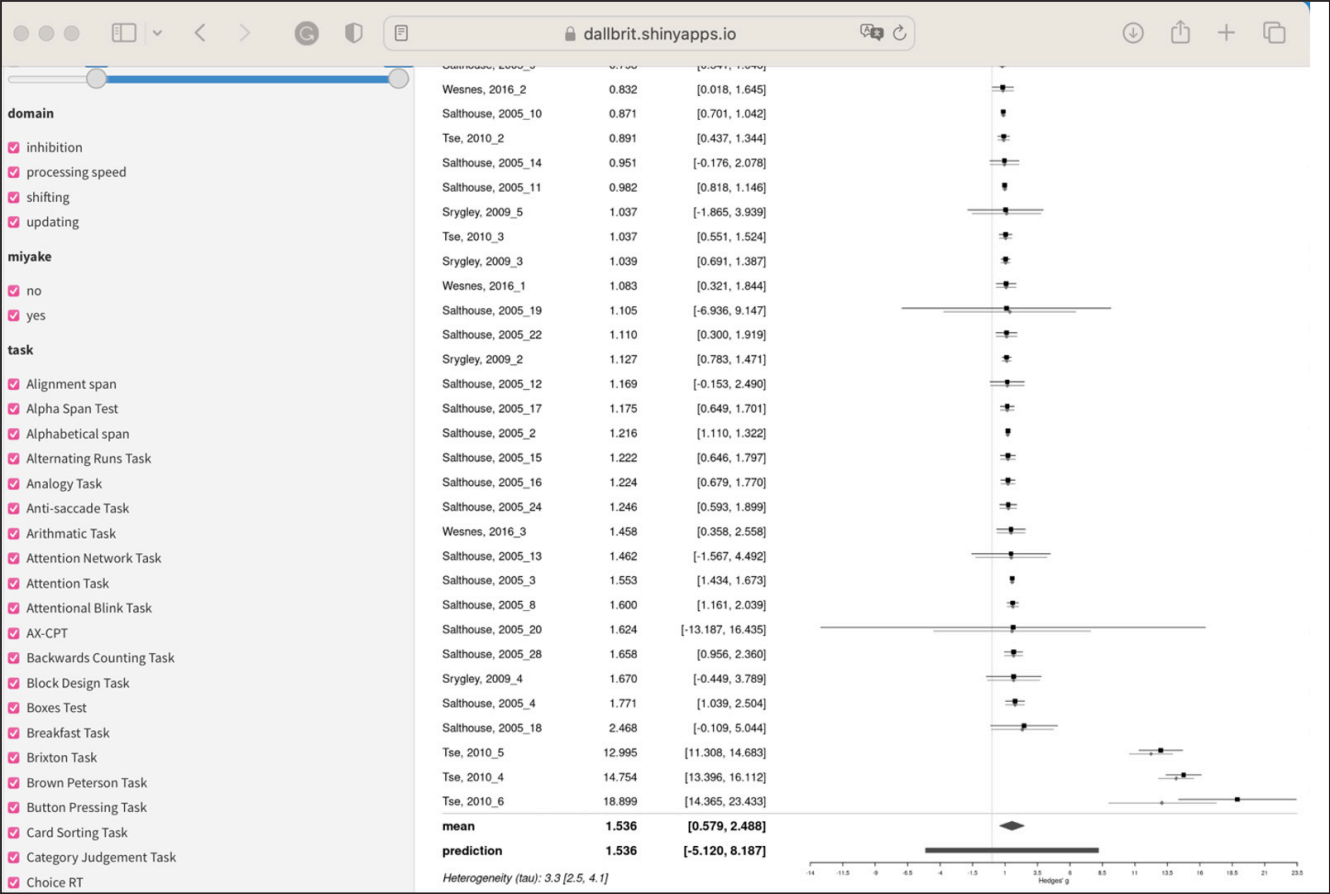
Forest plot of the papers from after 2005 in the Maldonado dataset and N > 80.

Given that larger Ns have become more common in recent years, we selected students with N > 80. There are 105 studies that meet the N and time of publication criterion. The mean for the posterior of the effect is 1.12 (larger than for the studies before 2005). Figure 11 shows the forest plot for this analysis.

#### Lexical and Reading Experiments

**Figure 12:**
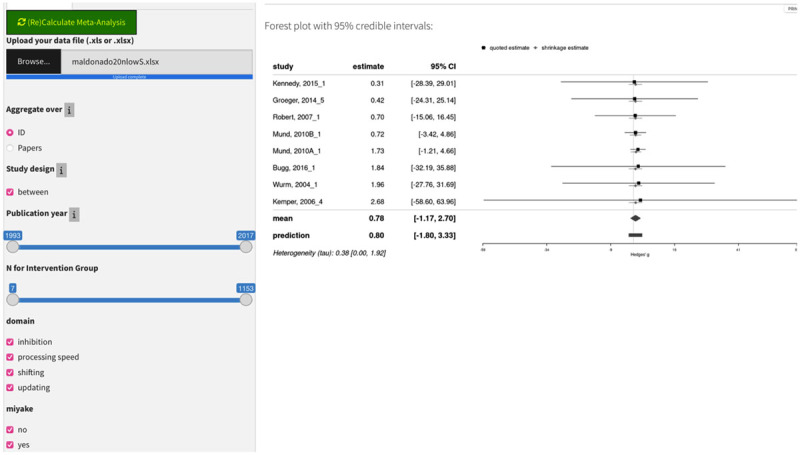
Forest plot of the lexical and reading-based studies in the Maldonado dataset.

It comes as no surprise to anybody that aging has sizable effects on many cognitive tasks. There is, however, one domain in which aging has a relatively small effect on performance according to some studies (e.g., Ratcliff et al., 2004): lexical processes. To examine the evidence within Maldonado et al.’s studies, we selected only the lexical and reading-related tasks. There are only seven studies of this type, and the Bayes Factor (BF) is near 1, meaning there is insufficient evidence to make any claim. This indicates there is future work to be done on this question. The forest plot is shown in Figure 12.

In short, the re-analysis of the Maldonado et al. data allowed us to explore variables in a novel way and examine how this field of research has changed over the years. Fortunately, studies now have larger Ns, and there are independent variables that gain and lose favor.

## Discussion

This paper aimed to contribute to developing meta-analytic research with a shift towards dynamic and living tools. Our work helps this evolution by introducing an enhanced app that fosters interactive exploration, incorporates new evidence, and allows for easy implementation of both frequentist and Bayesian meta-analyses. Tools like ours empower researchers, promote transparency and reproducibility, and facilitate the dynamic exploration of scientific phenomena.

The present tool can be utilized “as-is” if researchers are satisfied with the provided analyses via the provided link. However, it is important to note that researchers with knowledge of Shiny apps and relevant R packages can deploy their own versions. In both cases, our vision is to encourage groups or scholars to crowdsource and curate collections of studies on specific topics and post the data files in a different location like Google Sheets.

The technology required for this purpose is simple and widely available. For instance, tools like Google Sheets can easily be employed to maintain a database of studies to be meta-analyzed. Whether to make the dataset open for modification by anyone or secure it behind a password entails certain advantages and disadvantages that communities of scholars need to assess.

Given that Bayesian analyses are often time-consuming, we suggest the following workflow for maximum efficiency: once the desired family of studies is decided, the frequentist version of the analyses should be performed first for basic quality control and exploration of the studies, even if the Bayesian analyses are preferred by the researcher. Only once the researcher is certain that all data and parameters are entered correctly should the Bayesian calculations be performed.^2^

We should also stress that we do not aim to provide guidelines on how to perform a meta-analysis or any mechanism for assessing the quality and relevance of new studies (for this, see e.g., Egger et al., 2022). Those decisions need to be based on substantial expertise for which no app can substitute. We do advocate, on the other hand, for a standardized format for meta-analysis data sets so that the analyses proposed in this paper can be broadly available. The format proposed here is a good starting point, and in recent years, it has become evident that the field responds to guidelines positively (Giofrè et al., 2022).

### Suggestions for the future

Looking ahead, there is significant potential for advancing dynamic meta-analysis methodologies through crowdsourcing to gather relevant data, employing either a light or heavy curation approach. Our framework currently relies solely on Hedges *g* for effect size estimates and does not support mediator analysis or meta-regression models. However, incorporating these functionalities could be achieved quickly, as demonstrated by the availability of options such as *bayesmeta*. Users must be mindful of appropriately setting up contrast coding for the moderator variables. We believe that the current application can serve as a blueprint for forthcoming iterations, wherein all novel enhancements will be documented in separate folders accessible via an OSF link (https://osf.io/3zxh2/). These folders will include a concise explanation of the newly introduced features.

We hope that researchers publishing meta-analyses will use our tool (or its successors) to maintain, update, curate, and moderate the list of relevant studies. This could be achieved by creating a GitHub repository, which can be updated by the authors or forked by other contributors using well-established version-control procedures such as git. For example, Allotey et al. (2020) presented a meta-analysis on maternal and perinatal COVID-19, updating the included studies twice, with the online version of their paper reflecting these updates. Our tool would make such efforts significantly more feasible in psychology, as updates can be ongoing and do not have to wait for the original authors to produce a new version.

There could even be a badge for living meta-analysis papers and/or the option to publish an updated “snapshot” of the analysis as a pre-approved Registered Report to incentivize maintaining an up-to-date meta-analysis. A living and continuously updated meta-analysis could have a much stronger impact on a field of research than an aging, static meta-analysis. We envision that the curation of relevant studies, along with crowdsourcing (e.g., managed through GitHub and similar platforms), can balance openness and quality control. Users of such living meta-analyses could utilize the tool’s outputs to calculate plausible effect sizes, estimate priors, and develop new research questions.

## Data Accessibility Statement

The tool and the sample data are available at https://dallbrit.shinyapps.io/Breathing_Life_into_MetaAnalysis/ and at https://osf.io/3zxh2/.

## Footnotes

We thank Richard Morey (personal communication) for the classic references.

The Bayesian calculations can take a long time if there are many studies in the data set. For our default data file containing 79 effect sizes, for example, it took 504 seconds to run locally in RStudio on a Dell Inspiron 3793 with an Intel i7–1065G7 CPU and 16GB of RAM running Windows 11. Because any change in the study selection parameters requires recalculation, conducting an extensive sensitivity analysis takes even longer. For example, running the “Bayes factor robustness check” in the app on the same dataset took over 23 minutes.
